# The Engineered Synthesis and Enhancement of Nitrogen and Chlorine Co-Doped Fluorescent Carbon Dots for the Sensitive Detection of Quercetin

**DOI:** 10.3390/ma18112669

**Published:** 2025-06-05

**Authors:** Yuan Jiao, Xuewen Miao, Lizhang Wang, Shasha Hong, Yifang Gao, Xin Wang

**Affiliations:** 1 School of Environment and Spatial Informatics, China University of Mining and Technology, Xuzhou 221116, China; jiaoyuan@tyut.edu.cn (Y.J.); wlzh0731@cumt.edu.cn (L.W.); 2 Wanli Energy Technology Development Co., Ltd., Zhejiang Wanli University, Ningbo 315100, China; 3 College of Environment and Ecology, Taiyuan University of Technology, Jinzhong 030600, China; 16696243382@139.com; 4 Shanxi Institute for Functional Food, Shanxi Agricultural University, Taiyuan 030031, China; sshong@sxau.edu.cn

**Keywords:** nitrogen, chlorine co-doped, red emission carbon dots, quercetin, environmental safety monitoring

## Abstract

Flavonoid alcohols, particularly quercetin, as emerging antioxidants, demand advanced detection methodologies to comprehensively explore and evaluate their potential environmental and health risks. In this study, nitrogen–chlorine co-doped carbon dots (N, Cl-CDs), featuring an extended wavelength emission at 625 nm, were synthesized via the reaction of 4-chloro-1,2-phenylenediamine with polyethyleneimine. The engineered N, Cl-CDs exhibit superior photostability, exceptional aqueous dispersibility, and anti-interference capability in complex matrices. Leveraging static electron transfer mechanisms, the N, Cl-CDs demonstrate selective fluorescence quenching toward quercetin with an ultralow detection limit of 60.42 nM. Validation through rigorous spiked recovery assays in apple peel and red wine has been proficiently performed with satisfactory accuracy, highlighting the significant prospect of the constructed N, Cl-CDs for quercetin identification in real samples. This study provides valuable insights into the analytical determination of flavonoid compounds in complex environmental matrices, highlighting the potential of N, Cl-CDs for environmental and food safety monitoring.

## 1. Introduction

Quercetin, also known as oak phenol, is a polyphenolic flavonoid that exhibits a broad range of biological activities [1,2] and is abundantly found in vegetables, fruits, and traditional medicine [3]. It has garnered significant attention due to its pharmacological properties, including antioxidant [4,5], anti-inflammatory [6,7], antidiabetic [8], and anticancer [9,10,11] activity, as well as its potential in combating cardiovascular and cerebrovascular diseases [12]. Despite its therapeutic benefits, recent toxicological studies have indicated that quercetin, at elevated concentrations, may exhibit mutagenic effects on human genetic material [13]. As a result, the accurate detection of quercetin is essential for advancing pharmacological, biological, and toxicological research.

Numerous methods have been established to identify quercetin, such as Raman spectroscopy [14], HPLC (high-performance liquid chromatography) [15], and electrochemical techniques [16]. While these methods offer excellent sensitivity and precision, they are often hindered by drawbacks such as the need for expensive equipment, complex procedures, and the extensive use of organic solvents. Consequently, there is a pressing need to establish a cost-effective, efficient, and environmentally friendly approach for quercetin detection. Fluorescence-based detection has gained significant attention as a preferred technique, attributed to its quick results, simplicity, affordability, and high sensitivity [17,18,19].

Carbon dots (CDs) have attracted considerable attention in recent years due to their stable fluorescence [20,21,22], low toxicity [23,24,25], excellent biocompatibility [26,27,28], and ease of surface modification [29,30,31]. These advantageous properties make CDs highly promising candidates for fields including cellular imaging and fluorescence-based sensing. To enhance their fluorescence performance, Doping approaches have enabled the integration of diverse heteroatoms (e.g., N, S, B, P, and halogens) into the framework of CDs [32,33]. For instance, Liu et al. [34] synthesized nitrogen-doped blue light-emitting carbon dots (N-CDs) via a simple hydrothermal reaction between polyethyleneimine and anhydrous citric acid, subsequently developing an on–off fluorescence sensor for the efficient and reliable determination of Hg^2+^ and I^−^ ions in environmental matrices.

However, for CDs doped with a single element, the emission wavelength is often restricted, limiting their versatility [35]. Research has demonstrated that co-doping with two or more different elements can significantly enhance the optical properties of CDs [36,37]. The synergistic effects between the dopant atoms can alter the electronic structure of the CDs, thereby improving their fluorescence performance. For example, Kaleem et al. [38] investigated the influence of nitrogen and sulfur doping using thiourea and glutathione as molecular precursors, highlighting the role of precursor-directed ground-state heterogeneity in modulating the fluorescence emission characteristics of CDs. Therefore, the effective doping of CDs and the tailoring of their optical properties are of considerable research significance for the development of fluorescence-based sensing methods for quercetin detection.

**Scheme 1:**
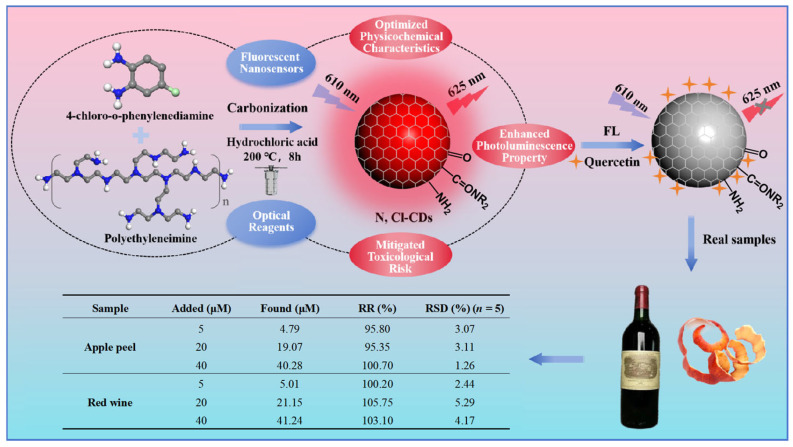
A schematic depiction of the synthesis process and diverse applications of N, Cl-CDs.

Taking into account the above limitations, nitrogen–chlorine-doped fluorescent carbon dots (N, Cl-CDs) with an emission wavelength of 625 nm are facilitated by the synergistic reaction between 4-chloro-o-phenylenediamine and polyethyleneimine. The distinct, concentration-dependent fluorescence response of N, Cl-CDs was observed upon interaction with quercetin, showing a linear quenching effect across the concentration ranges of 0.2–5 μM and 5–40 μM. Leveraging this unique quenching phenomenon, an innovative fluorescence-based sensing platform was devised for the precise quantification of quercetin. To further substantiate the dependability of the constructed method, spiked recovery experiments were conducted using red wine and apple peel samples, yielding outstanding recovery rates. The proposed fluorescence sensing strategy holds immense promise for food safety and environmental monitoring, providing a highly robust and precise analytical tool for the detection of flavonoid compounds (Scheme 1).

## 2. Materials and Methods

### 2.1. Preparation of N, Cl-CDs

Specifically, 0.500 g of polyethyleneimine and 0.108 g of 4-chloro-o-phenylenediamine were dissolved in 20 mL of ultrapure water. To facilitate the reaction, 1 mL of concentrated hydrochloric acid was introduced, and the mixture was subjected to ultrasonic dispersion. The resulting mixture was then transferred to a 50 mL autoclave and heated at 200 °C for 8 h. Upon the completion of the reaction, the solution was centrifuged at 10,000 rpm for 10 min to separate the supernatant. The product was dialyzed against ultrapure water choosing a membrane with a molecular weight cut-off of 0–500 Da and purified through a 0.22 μm filter. Ultimately, the purified product was freeze-dried to obtain the solid N, Cl-CDs.

### 2.2. Detection of Quercetin Based on N, Cl-CDs

In a routine procedure, the prepared N, Cl-CDs powder was dissolved in ultrapure water to obtain a concentration of 1.0 mg·mL^−1^. Subsequently, 80 µL of N, Cl-CDs solution was combined with 2.0 mL of PBS solution (pH = 7.0), and varying concentrations of quercetin were introduced into the prepared N, Cl-CDs dispersion. The fluorescence emission spectrum for each sample was measured at an excitation wavelength of 610 nm. The interference effects of other chemicals were individually assessed in the presence of N, Cl-CDs, following the same procedures as the quercetin. All experimental procedures were repeated in triplicate to ensure the reproducibility and reliability of the results.

### 2.3. Actual Sample Preparation

The apples utilized in this study were procured from a local supermarket. The apple peels were carefully removed using a fruit knife, and the resulting peel was sectioned into rectangular samples approximately 2 cm × 1 cm in size. These peel samples were then subjected to drying in an oven for 24 h until a constant weight was achieved. Subsequently, a 1 g portion of the dried apple peel was transferred to a beaker, and 10 mL of ethanol was added to extract the organic compounds present in the peel. The mixture was left to soak for 12 h to facilitate the extraction process. Following extraction, 50 μL of the apple peel extract was mixed with 2 mL of phosphate-buffer solution (pH = 7.0) and 80 μL of the prepared N, Cl-CDs. The mixture was placed in a 10 mm quartz cuvette and allowed to equilibrate at ambient temperature for 2 min. The fluorescence emission at 625 nm was measured following excitation at 610 nm.

The red wine for this experiment was purchased from a local store. The sample preparation procedure was as follows: 20 mL of the red wine was transferred into centrifuge tube and centrifuged at 10,000 rpm for 10 min to isolate the supernatant. Subsequently, 50 μL of the red wine supernatant, 2 mL of PBS solution (pH = 7.0), and 80 μL of the synthesized N, Cl-CDs were added into a 10 mm quartz cuvette. Following a 2 min incubation at ambient temperature, the fluorescence signal at 625 nm was detected upon excitation at 610 nm. Experimental validation included tripartite verification processes throughout the methodology to confirm result consistency.

## 3. Results and Discussion

### 3.1. Characterization of N, Cl-CDs

**Figure 1:**
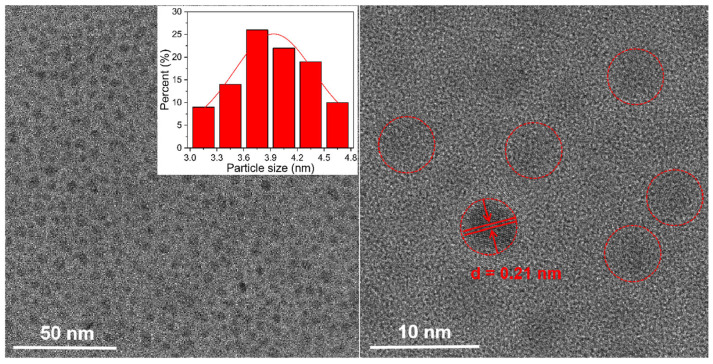
TEM images of the N, Cl-CDs including their size distribution in the top-right inset and the HR-TEM details.

Transmission electron microscopy (TEM) was employed to characterize the morphology of N, Cl-CDs. As depicted in Figure 1, the N, Cl-CDs exhibit a nearly spherical shape and are uniformly dispersed. Statistical analysis of 100 individual particles via Gaussian fitting revealed a narrow size distribution with an average diameter of 3.94 ± 0.88 nm, confirming their near-monodisperse characteristic. The high-resolution TEM (HRTEM) image in the inset of Figure 1 reveals well-defined crystalline structures within the N, Cl-CDs, displaying a lattice spacing of 0.21 nm, which is attributed to the (100) crystallographic plane of graphite-like carbon.

**Figure 2:**
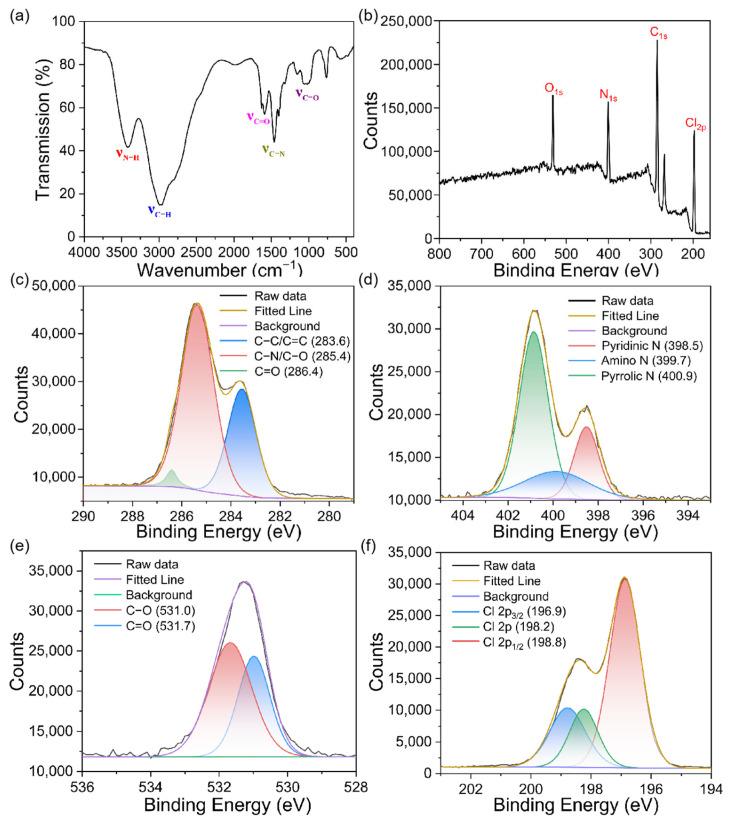
(**a**) FT-IR spectra, (**b**) XPS survey scan, and high-resolution XPS spectra of N, Cl-CDs, emphasizing the binding energy regions corresponding to (**c**) C_1s_, (**d**) N_1s_, (**e**) O_1s_, and (**f**) Cl_2p_.

Surface functional groups and elemental composition profiles were established through complementary Fourier-transform infrared spectroscopy (FTIR) and X-ray photoelectron spectroscopy (XPS). As depicted in Figure 2a, the FTIR spectrum reveals an absorption peak at 3417 cm^−1^, associated with the stretching vibrations of N-H. While the signals at 2991 cm^−1^ and 2964 cm^−1^ are linked to C-H stretching. The sharp peaks at 1597 cm^−1^ and 1400 cm^−1^ are attributed to the -C=O and -C-N= stretching vibrations, respectively. Further elemental analysis of the N, Cl-CDs was performed via XPS. Figure 2b shows that the N, Cl-CDs predominantly consist of four elements: C, N, O, and Cl, with atomic percentages of 58.36%, 18.45%, 10.45%, and 12.74%, respectively. These correspond to the characteristic peaks observed in the XPS spectra for C_1s_ (284.9 eV), N_1s_ (400.5 eV), O_1s_ (531.4 eV), and Cl_2p_ (197.4 eV). High-resolution XPS deconvolution in Figure 2c–f using Avantage 5.948 software revealed the surface chemical states of N, Cl-CDs: C_1s_ (283.6 eV: C=C/C-C, 285.4 eV: C-N/C-O, 286.4 eV: C=O), N_1s_ (398.5 eV: Pyridine N, 399.7 eV: Amino N, 400.9 eV: pyrrolic N), O_1s_ (531.0/531.7 eV: C-O/C=O), and Cl_2p_ (196.9 eV: Cl_2p_3/2, 198.2 eV: Cl_2p_, 198.8 eV: Cl_2p_1/2). Based on the FTIR and XPS analysis, it is concluded that the N, Cl-CDs are composed predominantly of C, N, O, and Cl elements. The carbon backbone is constructed from π-conjugated structures, while the surface is rich in amino- and chlorine-containing functional groups, indicative of their potential for various applications in optoelectronics and catalysis.

### 3.2. Optical Characteristics of N, Cl-CDs

**Figure 3:**
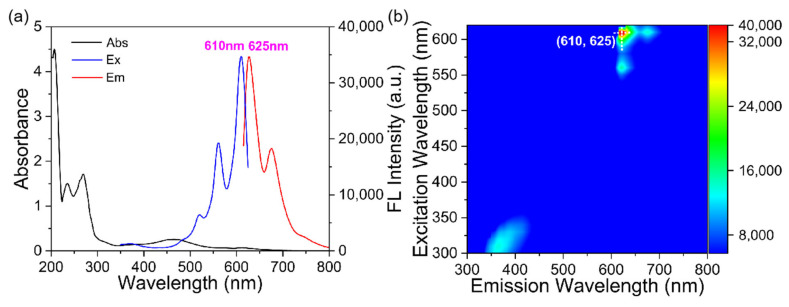
(**a**) UV–Vis absorption and fluorescence spectra of N, Cl-CDs; (**b**) excitation–emission matrix for N, Cl-CDs.

The optical properties of the R-CDs were assessed by UV–Vis absorption and fluorescence spectra. As depicted in Figure 3a, the peaks at 269 nm and 463 nm are ascribed to the π-π* transition of aromatic C=C bonds and the n-π* transition of C=O or C=N functional groups. Furthermore, the fluorescence spectrum reveals that the optimal excitation (λex) and emission (λem) wavelengths of N, Cl-CDs are 610 nm and 625 nm, respectively. Figure 3b further illustrates that the emission wavelength of N, Cl-CDs remains consistent across an excitation range of 300 nm to 620 nm, suggesting the uniform surface properties of the N, Cl-CDs. Furthermore, the calculated quantum yield for N, Cl-CDs was 20.6%, with rhodamine B serving as the standard reference (Figure S1).

The photoluminescent properties of N, Cl-CDs were further explored under various conditions to gain a deeper understanding of their behavior and stability. Photobleaching resistance evaluation under xenon irradiation revealed sustained emission at 625 nm, with <5% intensity fluctuation over 60 min illumination (Figure S2). Moreover, a slight decrease in fluorescence intensity is observed as the NaCl concentration increases (0–2 mol·L^−1^), demonstrating the excellent salt tolerance of N, Cl-CDs (Figure S3). The N, Cl-CDs demonstrates pH-responsive characteristics compatible with biological environments, with fluorescence intensity reaching a plateau in the physiological pH range (6.0–7.0) and showing a bidirectional attenuation trend under non-physiological pH conditions (Figure S4). These findings emphasize the significant potential of N, Cl-CDs in various sensing applications, underscoring their stability and versatility in diverse environmental conditions.

### 3.3. Fluorescent Behavior of N, Cl-CDs in Response to Quercetin

**Figure 4:**
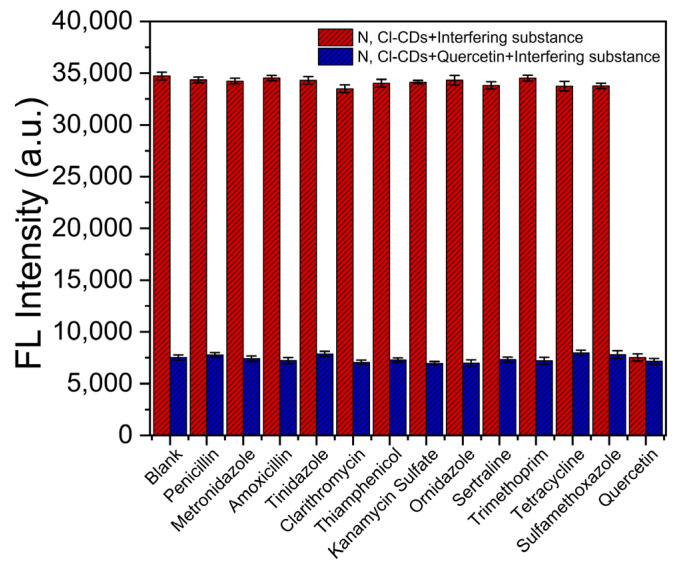
The selectivity evaluation of N, Cl-CDs in response to a diverse range of antibiotics and flavonoid compounds.

To investigate the specific interaction between N, Cl-CDs and quercetin, a fluorescence intensity analysis was conducted by incubating N, Cl-CDs with various antibiotics and flavonoid compounds, including penicillin, metronidazole, amoxicillin, tinidazole, clarithromycin, thiamphenicol, kanamycin sulfate, ornidazole, sertraline, trimethoprim, tetracyclin, sulfamethoxazole, and quercetin. Upon adding 5 μL of each compound (at a concentration of 0.01 mol·L^−1^) to the N, Cl-CDs solution, the fluorescence emission at 625 nm exhibited negligible changes. In contrast, a rapid and pronounced decrease in fluorescence intensity was recorded exclusively upon the addition of quercetin, emphasizing the specific and highly sensitive reaction to quercetin of N, Cl-CDs (Figure 4).

**Figure 5:**
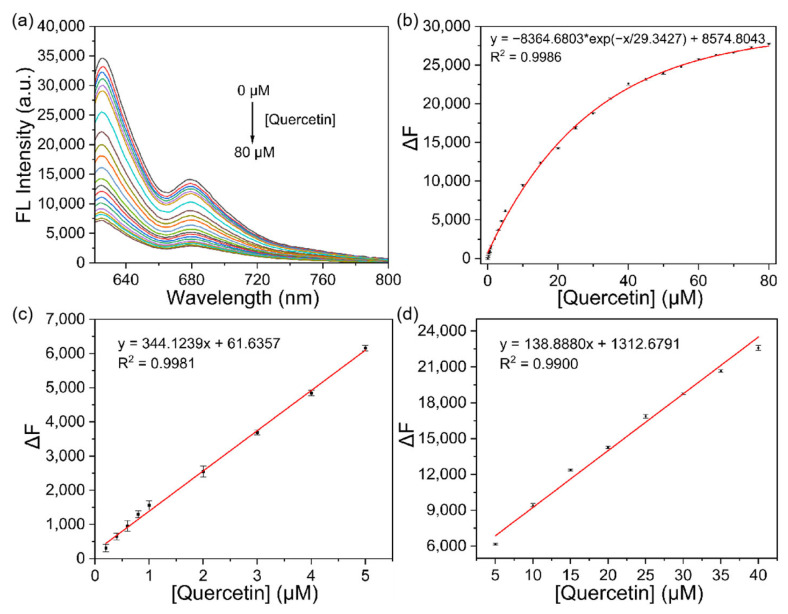
(**a**) Fluorescence spectra of R-CDs following the addition of quercetin; (**b**) fluorescence intensity of N, Cl-CDs at 625 nm as a function of quercetin concentration; (**c**,**d**) corresponding linear relationships between fluorescence intensity and quercetin concentration.

Taking physiological conditions into account, pH 7.0 was chosen as the optimal environment thanks to the significant optical performance of N, Cl-CDs towards this pH. Upon quercetin introduction, pH 7.0 demonstrates the most substantial reduction in fluorescence, in contrast to the diminished intensity observed under acidic and alkaline conditions. These experimental findings collectively confirm that pH 7.0 represents the optimal condition for achieving both high sensitivity and robust stability. As depicted in Figure 5, the N, Cl-CDs based fluorescence sensor demonstrates excellent quercetin responsiveness. Upon the incremental addition of quercetin, the fluorescence emission intensity at 625 nm exhibits a concentration-dependent suppression, reaching near-complete quenching at 80 μM. The quantitative analysis of fluorescence intensity changes reveals a significant linear correlation within the concentration ranges of 0.2–5 μM and 5–40 μM. The experimental data are optimally fitted to an exponential decay function (ExpDec1 model), yielding two linear regression equations: ΔF = 344.1239x + 61.6357, R^2^ = 0.9981 and ΔF = 138.8880x + 1312.6791, R^2^ = 0.9900 (ΔF = F_0_ − F, where F_0_ and F denote the fluorescence intensity of N, Cl-CDs at 625 nm before and after the introduction of quercetin, respectively). The limit of detection (LOD) for quercetin is determined to be 60.42 nM.

### 3.4. Recovery of Quercetin from Actual Samples

To evaluate the prospective utilization of N, Cl-CDs in food safety analysis, the recovery efficiencies of quercetin were systematically examined in apple peel and red wine, as summarized in Table 1. Quercetin at various concentrations (5, 20 and 40 μM) was spiked into the apple peel and red wine samples. In the apple peel samples, recovery rates ranged from 95.35% to 100.70% (RSD ≤ 3.11%), while in the red wine samples, recovery rates varied from 100.20% to 105.65% (RSD ≤ 5.29%). These findings underscore the potential of N, Cl-CDs as a highly reliable and accurate analytical tool, demonstrating significant promise for the precise quantification of quercetin in complex food matrices.

### 3.5. Sensing Mechanism of Quercetin by N, Cl-CDs

**Figure 6:**
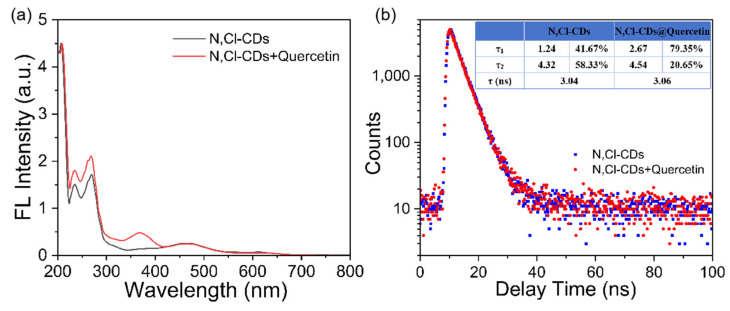
(**a**) The UV–Vis absorption spectra of N, Cl-CDs, along with the incorporation of quercetin; (**b**) the fluorescence decay lifetime of N, Cl-CDs in the presence and absence of quercetin and related data.

To further investigate the sensing mechanism of N, Cl-CDs towards quercetin, a comprehensive analysis was conducted using UV–visible absorption spectroscopy and fluorescence lifetime measurements. The UV–Vis absorption spectra of N, Cl-CDs were initially recorded from 200 nm to 800 nm, with the baseline spectrum (represented by the black curve in Figure 6a) serving as a reference for subsequent comparison. Upon the addition of quercetin, a distinct and significant absorption peak appeared at 367 nm, indicating a strong and specific interaction between the N, Cl-CDs and the quercetin. Furthermore, fluorescence lifetime measurements were conducted to offer a more detailed understanding of the underlying interaction dynamics. The fluorescence lifetime of N, Cl-CDs exhibited remarkable stability, with only a subtle shift from 3.04 ns to 3.06 ns upon the introduction of quercetin (Figure 6b). This negligible change in fluorescence lifetime serves as a compelling indicator that the quercetin interaction predominantly follows a static quenching mechanism, as opposed to dynamic processes such as collisional quenching. This observation unequivocally underscores that static quenching, mediated by the formation of a ground-state complex between the fluorophore and the quencher, constitutes the principal mechanism through which quercetin orchestrates fluorescence modulation in N, Cl-CDs.

## 4. Conclusions

In conclusion, carbon dots co-doped with nitrogen and chlorine, emitting at 625 nm, have been innovatively synthesized for the precise detection of quercetin. The remarkable photostability and enhanced photoluminescence efficiency of the constructed N, Cl-CDs stand out as critical advantages for the application in environmental monitoring. Notably, the engineered N, Cl-CDs exhibit exceptional sensitivity as a fluorescence-based platform for accurate quercetin detection, displaying a marked fluorescence quenching response. Upon optimization, the fluorescence response of N, Cl-CDs exhibits a well-defined linear relationship with quercetin concentrations in the ranges of 0.2–5 μM and 5–40 μM, accompanied by an impressively low detection limit of 60.42 nM. The effectiveness of N, Cl-CDs for detecting quercetin residues in real-world samples, including apple peel and red wine, is further validated by the spiked recovery analysis. These results underscore the significant potential of the N, Cl-CDs based fluorescence sensing platform for environmental monitoring applications, offering a reliable and precise method for quercetin quantification in complex food matrices.

## Figures and Tables

**Table 1:** Detection of quercetin in apple peel and red wine samples based on N, Cl-CDs.

Sample	Added (μM)	Found (μM)	RR (%)	RSD (%) (*n* = 5)
**Apple peel**	5	4.79	95.80	3.07
	20	19.07	95.35	3.11
	40	40.28	100.70	1.26
**Red wine**	5	5.01	100.20	2.44
	20	21.15	105.75	5.29
	40	41.24	103.10	4.17

## Data Availability

The original contributions presented in this study are included in the article/Supplementary Materials. Further inquiries can be directed to the corresponding authors.

## Supplementary Materials

The following supporting information can be downloaded at: https://www.mdpi.com/article/10.3390/ma18112669/s1, Figure S1: Plots of integrated PL intensity against absorbance of (a) rhodamine in ethanol and (b) N, Cl-CDs solution at a λ_ex_ of 495 nm and relevant data; Figure S2: Time-dependent changes in the fluorescence intensity of N, Cl-CDs. The fluorescence intensity of N, Cl-CDs was monitored under continuous xenon arc lamp irradiation for 60 min, with data collected every second; Figure S3: The influence of ionic strength on the fluorescence intensity of N, Cl-CDs, λex/λem = 610 nm/625 nm; Figure S4: Fluorescence spectra of N, Cl-CDs and quercetin-added N, Cl-CDs over a pH range from 1 to 13.
