# Supplementary material for: The Engineered Synthesis and Enhancement of Nitrogen and Chlorine Co-Doped Fluorescent Carbon Dots for the Sensitive Detection of Quercetin
Source: Materials (Basel). 2025 Jun 5;18(11):2669. doi: 10.3390/ma18112669 (PMC12155965; doi:10.3390/ma18112669)
Supplement: Supplementary file 1 [file materials-18-02669-s001.zip › materials-3643302-supplementary.pdf]

Supplementary Material

The Engineered Synthesis and Enhancement of Nitrogen and Chlorine Co-Doped Fluorescent Carbon Dots for the Sensitive Detection of Quercetin

Yuan Jiao [1,2,3], Xuewen Miao [3], Lizhang Wang [1], Shasha Hong [4], Yifang Gao [1,*] and Xin Wang [2,*]

[1] School of Environment and Spatial Informatics, China University of Mining and Technology, Xuzhou 221116, China; jiaoyuan@tyut.edu.cn (Y.J.); wlzh0731@cumt.edu.cn (L.W.)
[2] Wanli Energy Technology Development Co., Ltd., Zhejiang Wanli University, Ningbo 315100, China
[3] College of Environment and Ecology, Taiyuan University of Technology, Jinzhong 030600, China; 16696243382@139.com
[4] Shanxi Institute for Functional Food, Shanxi Agricultural University, Taiyuan 030031, China; sshong@sxau.edu.cn
* Correspondence: gaoyifang@tyut.edu.cn (Y.G.); wangx@zwu.edu.cn (X.W.)

Experimental

Reagents

4-chloro-o-phenylenediamine and polyethyleneimine were obtained from Shanghai Aladdin Bio-Chem Technology Co., Ltd. Quercetin and related interfering substances were sourced from Shanghai Macklin Biochemical Co., Ltd. Ultrapure water (≥18.25 MΩ·cm) was prepared by the Rephile Genie water system and employed throughout all experimental procedures.

Instrumentation and Characterization

Fourier transform infrared (FT-IR) spectra of N, Cl-CDs were recorded with a PerkinElmer Spotlight 400 spectrometer (Waltham, Massachusetts, USA) covering a range of 4000 to 400 cm^{-1}. Transmission electron microscopy (TEM) images were captured by an F200 transmission electron microscope (Tokyo, Japan). X-ray photoelectron spectroscopy (XPS) data were acquired on a Thermo Scientific ESCALAB Xi+ system (New York, USA). Fluorescence spectra were measured in a Shimadzu RF-6000 fluorescence spectrophotometer (Tokyo, Japan). UV-Vis absorption spectra of N, Cl-CDs were recorded on a UV-8000s spectrophotometer (Shanghai, China). The nanosecond fluorescence lifetime was determined by an FLS 980 spectrofluorometer (Edinburgh, Livingston, UK). pH adjustments were precisely performed in the LE438 digital pH meter (Zurich, Switzerland).

Quantum yield of N, Cl-CDs

The quantum yield of N, Cl-CDs was determined by a comparative method:

$$\Phi_x = \Phi_b \left(\frac{Grad_x}{Grad_b}\right)\left(\frac{{\eta_x}^2}{{\eta_b}^2}\right)$$

The subscripts x and b denote the N, Cl-CDs sample and the reference (Rhodamine B in ethanol, QY = 89%), respectively. Grad represents the slope obtained from the plot of integrated fluorescence intensity against absorbance, and η signifies the refractive index of the solvent. For the measurement, a set of N, Cl-CDs solutions and reference samples were prepared with their concentrations adjusted to ensure that the optical absorbance intensity remained between 0 and 0.1 at 495 nm. The integrated fluorescence intensity was measured across a wavelength range of 515-800 nm.

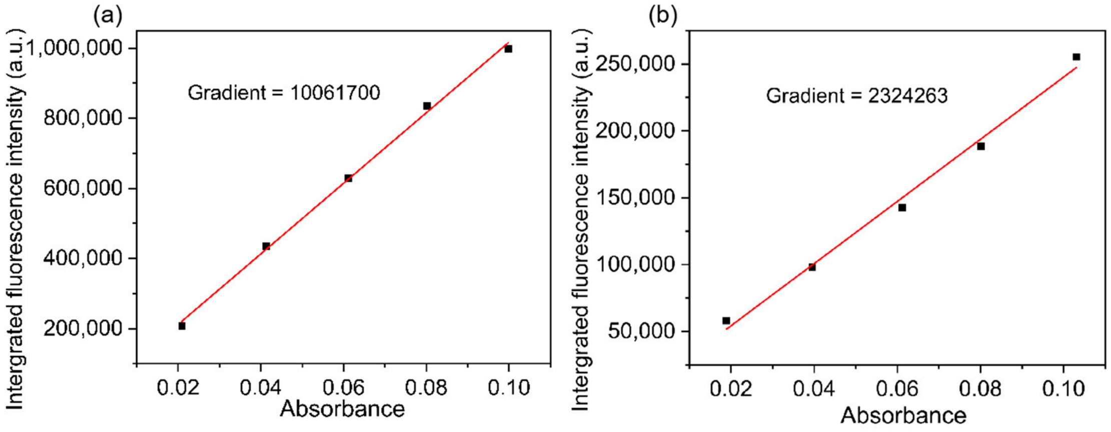

	Rhodamine B					N, Cl-CDs				
Abs	0.0209	0.0413	0.0611	0.0802	0.0998	0.0189	0.0395	0.0612	0.0801	0.1030
Integrated PL	208333	435489	628963	835693	997521	58162	98218	142549	188454	255479
Slope	10061700					2324263				
QY	89 %					20.6%				

Figure S1. Plots of integrated PL intensity against absorbance of (a) Rhodamine in Ethanol and (b) N, Cl-CDs solution at λ_{ex} of 495 nm and relevant data.

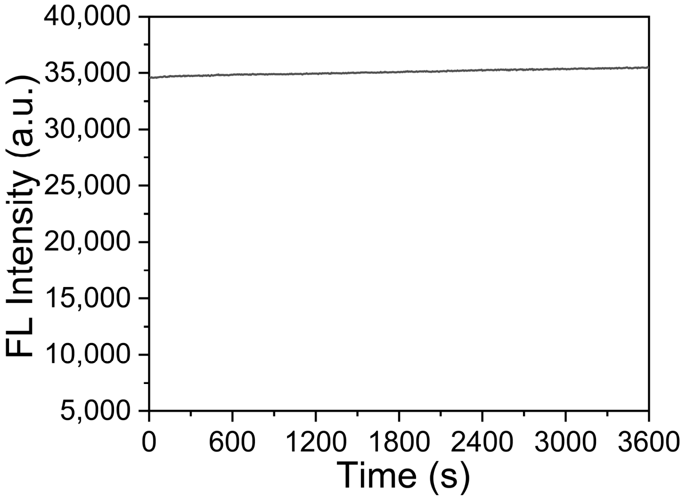

Figure S2. Time-dependent changes in the fluorescence intensity of N, Cl-CDs. The fluorescence

intensity of N, Cl-CDs was monitored under continuous xenon arc lamp irradiation for 60 min, with data collected every one seconds.

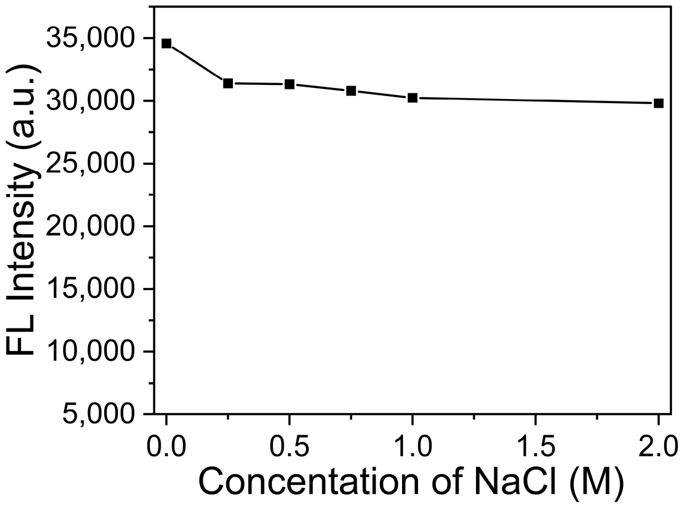

Figure S3. Influence of ionic strength on the fluorescence intensity of N, Cl-CDs, λex/λem = 610 nm/625 nm.

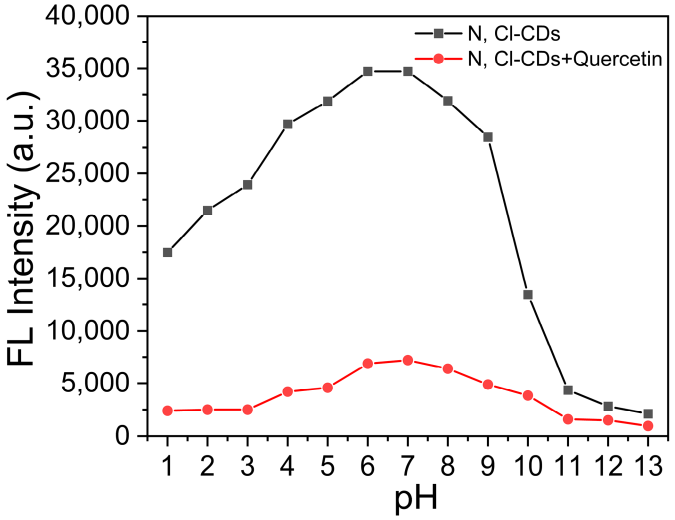

Figure S4. Fluorescence spectra of N, Cl-CDs and quercetin-added N, Cl-CDs over a pH range from 1 to 13.
